# ADH1B Gene Promotes Gastric Cancer Tumorigenesis and its Cisplatin Resistance through the EMT Pathway

**DOI:** 10.2174/0115680096351094241125111654

**Published:** 2025-02-26

**Authors:** Zhenguo Pan, Chengcheng Gao

**Affiliations:** 1 Department of Gastroenterology, The Affiliated Huaian No.1 People's Hospital of Nanjing Medical University, No. 1 Huanghe West Road, Huai'an, Jiangsu, 223300, China

**Keywords:** Gastric cancer, cisplatin, drug resistance, EMT, pathway immuno-histochemistry

## Abstract

**Background:**

Cisplatin resistance significantly affects the outcome of gastric cancer treatment. In this study, genes associated with cisplatin resistance were investigated and discussed.

**Methods:**

We analyzed the sequencing data of GSE14208 patients from the GEO database using differential and enrichment analyses. Gastric cancer cells with high ADH1B expression and low ADH1B expression were selected by qPCR and WB to construct ADH1B overexpress and silence cells. The optimal cisplatin concentrations for treatment were determined via CCK-8 detection and WB. Furthermore, we assessed drug resistance, cellular activity, and invasion and migration capacities using IC_50_, CCK-8 assays, Transwell, and migration tests. Apoptosis was evaluated using flow cytometry and EDU staining. The pathway influenced by ADH1B was examined through WB and immunofluorescence. The impact of gene expression on the tumorigenic potential of gastric cancer cells was assessed by analyzing tumor size, mass, and histology in HE-stained tumor sections and Ki67 and protein pathway immunohistochemistry in a mouse tumorigenesis model.

**Results and Discussion:**

This study revealed that ADH1B may exhibit a cancer-promoting effect, according to our database analysis, and is associated with drug resistance. Silencing ADH1B in AGS cells led to a reduced IC_50_, as well as decreased viability, invasion, and migration capabilities while increasing apoptosis rates. Conversely, overexpressing ADH1B in MKN-45 cells reversed these effects. Western blot and immunofluorescence results indicated that the expression levels of proteins involved in the EMT, PI3K, and MAPK pathways were altered following the silencing and overexpression of ADH1B. Moreover, silencing ADH1B not only reduced tumor volume and weight but also enhanced the tumor-reducing effects of cisplatin, as evidenced by changes in tumor structure; overexpression had the opposite effects. The alterations in pathway protein expression in tumor sections mirrored those observed in cells.

**Conclusion:**

This study identified the gene ADH1B as a critical factor in the incidence and drug resistance of gastric cancer. It was demonstrated through cellular and tumorigenic assays that ADH1B promotes carcinogenesis and enhances cisplatin resistance in gastric cancer cells *via* the EMT signaling pathways.

## 1 INTRODUCTION

Gastric cancer is a common type of gastrointestinal tumor that arises from the epithelial cells of the stomach lining [1]. It is recognized as a global disease characterized by a high degree of molecular and phenotypic heterogeneity [2-3]. Due to the absence of prominent early symptoms, unclear clinical indications, and low detection rates through routine screening, most patients receive their diagnoses at later stages of the condition [1-2]. Currently, surgical intervention remains the most significant clinical treatment. Chemotherapy remains the preferred option for perioperative treatment, as well as for adjuvant therapy and palliative care, in patients diagnosed with advanced gastric cancer. Patients, who generally have low survival rates, with cisplatin being one of the commonly utilized chemotherapeutic agents, are particularly those in advanced stages. The effectiveness of chemotherapy in improving the survival rates of patients with gastric cancer has been demonstrated [4].

Cisplatin is a non-specific cytotoxic drug that serves as the first-line treatment for many solid tumors and has been utilized in clinical practice for decades. It inhibits tumor cell viability and progression by inducing cellular DNA damage. Although cisplatin exhibits anticancer activity across a variety of tumors, its therapeutic efficacy, as well as patient prognosis, is influenced by toxicity and drug resistance [5]. The most significant challenge in cisplatin treatment remains resistance, primarily attributed to the overactivation of drug efflux pumps, gene dysregulation, and interactions within the tumor microenvironment. Currently, resistance to cisplatin may be associated with three molecular mechanisms: elevated DNA repair, modified cellular accumulation, and augmented drug inactivation [6-8].

The transformation of epithelial cells into mesenchymal cells occurs through a distinct program known as epithelial-mesenchymal transition (EMT) [9]. Throughout this process, cells shed their epithelial properties and structural integrity as they gain mesenchymal features [10]. The EMT pathway can modify intercellular adhesion and the extracellular matrix, leading to tumor cell invasion. This process results in significant changes in morphology and motility, contributing to both tumor cell invasion and chemoresistance [11]. Furthermore, EMT may orchestrate various complementary cancer properties, including stemness, tumorigenicity, drug resistance, and the adaptation of tumor cells to changes in their microenvironment [9, 12-14]. Research has indicated a correlation between the progression of gastric cancer and EMT [15-17]. In a similar vein, resistance to cisplatin has also been connected to EMT [18-20]. Both short- and prolonged cisplatin treatment can induce tumor cell resistance to the drug. Cisplatin can activate cellular autophagy, NF-κB, classically activated macrophages (CAMs), and Ataxia telangiectasia mutated (ATM) pathways, contributing to the induction of EMT. Multiple complex and dynamic molecular pathways exist that link cisplatin resistance to the EMT pathway. Given that EMT mediates cisplatin resistance through more than one molecular pathway, efforts to enhance sensitivity should target multiple pathways [11].

The most well-known function of ADH1B is its involvement in alcohol metabolism, but recent studies have reported that ADH1B is associated with tumorigenesis and patient prognosis [21-38]. For example, ADH1B is able to promote the infiltration of ovarian cancer cells and increases the likelihood of residual lesions after surgery. In addition, ADH1B can be used as a prognostic indicator for hepatocellular carcinoma (LIHC) and pancreatic ductal adenocarcinoma (PDAC).

Building upon the aforementioned studies, this research investigated the cisplatin resistance gene associated with gastric cancer and examined the role of the identified ADH1B gene in gastric cancer cells through cellular assays and tumor formation experiments. This not only offers a novel approach to addressing cisplatin resistance encountered in the clinical treatment of gastric cancer but also elucidates the newly discovered function of the ADH1B gene within gastric cancer cells.

## 2 MATERIALS AND METHODS

### 2.1 Cell Culture

MKN-45 (Procell, CL-0292), SGC-7901 (Beyotime, C6795), NCL-N87 (ATCC, CRL-5822), and SNU-16 (ATCC, CRL-5974) cells were maintained in RPMI-1640 (Gibco, A5670701) complete medium, which included 10% fetal calf serum (Gibco, A5670701) along with 100 U/mL of penicillin-streptomycin (Gibco, A5670701). Meanwhile, AGS cells (CRL-1739) were grown in Ham's F-12K (Gibco, 21127030) complete medium, which also comprised 10% fetal bovine serum and 100 U/mL of penicillin-streptomycin.

### 2.2 Bioinformatics Analysis

Genes that have a significant correlation with human gastric cancer were identified through the Gene Expression Omnibus (GEO) database (GSE14208). An analysis was conducted on the relationship between the identified genes and epithelial-mesenchymal transition (EMT) utilizing the GEPIA database (http://gepia2.cancer-pku.cn/#index), which assesses the expression levels of ADH1B in various tissues. This analysis included the assessment of expression differences of ADH1B in various tissues and the overall survival (OS) of the identified genes from the TCGA database (https://cancergenome.nih.gov/). The dataset was then divided into groups classified as high-risk and low-risk according to the computed risk scores.

### 2.3 Quantitative RT-PCR (qPCR)

RNA was isolated from the target cells utilizing the Tiangen Universal Reagent (TIANGEN, DP424). Subsequently, gene expression levels were assessed using the One Step RT-qPCR Kit (Sangon, B639277), utilizing glyceraldehyde-3-phosphate dehydrogenase (GAPDH) as the reference gene to achieve normalization. The expression levels of the gene were determined through the 2-ΔΔCT calculation method.

### 2.4 Western Blotting (WB)

Cells were lysed with RIPA lysis buffer (P10013B, Beyotime), followed by quantification of protein concentration using the BCA Protein Quantification Kit. The proteins were then separated by SDS-polyacrylamide (SDS-PAGE) gel electrophoresis and subsequently transferred to membranes, and analyzed using the corresponding antibodies: ADH1B (Proteintech, 66939-1-Ig), E-cadherin (Proteintech, 20874-1-AP), N-cadherin (Abcam, ab76011), Vimentin (Proteintech, 10366-1-AP), p-JAK2 (Thermo, PA5-36658), JAK2 (Thermo, PA5-121814), p-Stat3 (Thermo, MA5-15193), GAPDH (Abcam, ab8245), and Stat3 (Thermo, 710077). The antibodies were allowed to incubate overnight at 4°C, and then on the next day, they were incubated with either Goat anti-Rabbit (Abcam, ab6721) or Goat anti-Mouse (Solabio, SE131) antibodies. Protein bands were visualized using a DAB kit (Beyotime, P0203).

### 2.5 Construction of AGS with Low Expression of ADH1B and MKN-45 Cell Overexpressing ADH1B

The si-ADH1B sequence was synthesized by Sangon (Shanghai, China). The si-ADH1B was transfected into AGS cells using Lipo 3000 (Invitrogen, L3000015) to establish AGS cells with reduced expression of ADH1B (si-ADH1B). The efficiency of ADH1B knockdown was confirmed using qPCR and WB. A stable AGS cell (shADH1B) with low expression of ADH1B was subsequently constructed. The shADH1B plasmid was also synthesized by Sangon (Shanghai, China). For the transduction process, 293T cells (ATCC, CRL-3216) were cultured in serum- and penicillin-strepto-mycin-free medium. The reaction mixture was created and slowly incorporated dropwise into the incubation medium. After a designated incubation period, the old medium was discarded, fresh medium was added, and the cell supernatant was collected after 48 hours of fluid exchange. This supernatant was then centrifuged at 4°C, passed through a 0.45 μm filter, and preserved at -80°C. AGS cells were inoculated into a culture dish, permitted to attach to the surface, and then the medium was replaced. Lentiviral infection was performed, followed by a medium change after 24 hours, continuing the culture for an additional 48 hours. After this period, fresh medium containing Puromycin was added to select stably transduced cells. For the construction of MKN-45 (oe-ADH1B) cells, the ADH1B gene was overexpressed for either 24 or 48 hours. The sequences of oe-ADH1B were generated by Tsingke Biotech Co. To evaluate the efficiency of ADH1B overexpression, we utilized qPCR and WB.

### 2.6 Cell Function Assay

The CCK8 assay and EdU proliferation assay were employed to assess cell proliferation capabilities. The CCK8 assay involved inoculating cells into 96-well plates, followed by the addition of cisplatin (Merck, 232120) and incubation. At the conclusion of the incubation period, The diluted CCK8 reagent (Beyotime, C0037) was introduced to every well, and the cells were incubated in the dark. The optical density (OD) was calculated using the formula: (ODcell - ODblank)/(ODcontrol - ODblank) × 100%. The EdU proliferation assay was conducted by adding cisplatin to the designated group after cell spreading, discarding the old solution, and replacing it with an appropriate amount of complete medium, in accordance with the instructions provided by the EdU Cell Proliferation Kit with DAB (Beyotime, C0085S). To assess the ability of the cells to invade, they were placed in the upper compartment of a Transwell filled with matrix gel (CORNING, 356237), with the complete medium containing cisplatin added to the lower wells. The cells in the lower wells were subsequently collected and counted under a microscope. For the cell scratch assay, which assessed cell migration ability, the cells were allowed to adhere to the culture dish, after which a pipette tip was used to vertically scratch the surface to remove floating cells. The change in the width of the scratch was measured and compared across each group. In the immunofluorescence assay, the old medium in the cell dish was discarded, and the cells were fixed with 4% paraformaldehyde. Following this, the cells were washed with PBS and incubated with the corresponding antibody. Following further washing with PBS, the cells were treated with a secondary antibody conjugated to HRP, subsequently washed with PBS once more, and then exposed to a DAPI solution for one minute. After discarding the DAPI solution, PBS was added, and the cells were examined using a fluorescence microscope. Ultimately, the levels of apoptosis were evaluated through the use of flow cytometry. Cells treated with cisplatin were collected after incubation and processed according to the kit instructions (Beyotime, C1062L), followed by analysis using Flowjo software.

### 2.7 Establishment of Mouse Tumor Model

Nude mice were acquired from the Guangdong Medical Laboratory Animal Center and placed in a standardized animal facility, with approval from the Animal Ethics Committee of Guangzhou Myers Biotechnology Co. for the study (IACUC-MIS20230058). A total of 5×106 cells were injected subcutaneously into 4-week-old nude mice, followed by the administration of cisplatin *via* the tail vein after tumor formation. Starting on the first day of treatment, the mice received injections every two days, and their weights were recorded over a consecutive 28-day period. On day 28, the mice were euthanized using an overdose of anesthesia, and the tumor tissue was subsequently excised.

### 2.8 HE Staining

After paraffin embedding of the tumor tissue, 4 μm thick sections were prepared. The tissues were treated with drops of hematoxylin staining solution and allowed to incubate for 5 minutes, after which they were rinsed with distilled water. The tissues were then incubated with Dako bluing buffer for 1 minute, washed again with distilled water, and subsequently stained with eosin solution for 1 minute. After another wash with distilled water, the sections were immersed in ethanol solutions of 75%, 85%, 95%, and 100% concentrations for 2-3 seconds in sequence. Finally, the sections were covered with anhydrous ethanol for 1 minute, treated with xylene for 1 minute twice, and sealed with tree resin after each treatment.

### 2.9 Immunohistochemistry

The samples were soaked in a 10 mM sodium citrate solution at a temperature of 95°C for a duration of 20 minutes. After removal, they were incubated dropwise with a 3% H2O2 solution for 10 minutes and subsequently washed three times with PBS. Next, diluted ki67 (Proteintech, 27309-1-AP), antibodies against E-cadherin, N-cadherin, and p-Star3 were introduced and left to incubate overnight at 4°C, after which three washes with PBS were performed. The sections were then washed three more times before adding the corresponding HRP-containing antibody and incubating for two hours. Ultimately, they underwent three wash cycles with PBS and were then processed utilizing the DAB technique.

### 2.10 Statistical Analysis

Data analysis was conducted with GraphPad Prism version 9.5.1 unless stated otherwise. The differences between the two groups were evaluated using one-way ANOVA, establishing a significance threshold of *p* < 0.05.

## 3 RESULTS

### 3.1 Screening of Differential Genes and Bioinformatics Analysis of their Functional Pathways

**Fig. (1):**
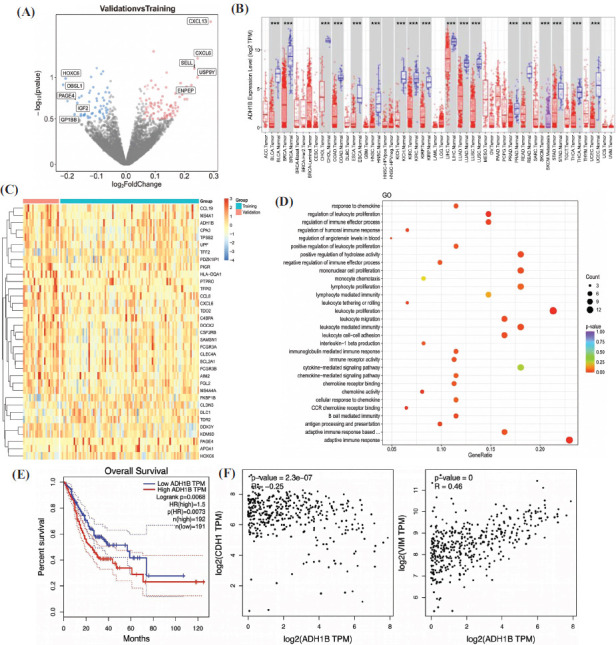
Results of biosignature analysis. (**A**, **C**): GEO database screening was conducted to identify differentially expressed genes, with a heatmap illustrating the top 35 such genes. (**B**): The distribution of ADH1B expression across various tumor tissues compared to normal tissues was analyzed. (**D**): An analysis of the biological processes associated with the differentially expressed genes was performed. (**E**): The overall survival analysis related to ADH1B was conducted. (**F**): A correlation analysis of ADH1B with E-Cadherin (CDH1) and Vimentin (VIM) was undertaken.

The comparison of results from GSE14208 in the GEO database revealed multiple up- and down-regulated differential genes. The heatmap depicted the 35 most significantly differentially expressed genes when comparing gastric cancer patients to the normal group (Fig. **1A** and **C**). The findings from the Gene Ontology (GO) analysis emphasized the roles of the identified differentially expressed genes (DEGs), which played a part in regulating leukocyte proliferation, their movement, adhesion, signaling pathways mediated by cytokines, and processes related to immune effectors (Fig. **1D**). Box plots demonstrated the expression of ADH1B across various tumor and normal tissues (Fig. **1B**). Furthermore, Kaplan-Meier curves were generated using the TIM database to investigate survival differences between high and low expression levels of ADH1B (Fig. **1E**). Survival analysis, utilizing risk ratios and the Log-rank test, revealed that patients exhibiting high ADH1B expression had a considerably reduced overall survival rate compared to those with low expression levels. Correlation analysis of ADH1B with drug resistance-related genes CDH1 and VIM showed that ADH1B was negatively correlated with CDH1 (R = -0.25, P =2.3e-07) and positively correlated with VIM (R = 0.46, P = 0) (Fig. **1F**).

### 3.2 Expression Difference of ADH1B in Different Gastric Cancer Cells and Cisplatin Treatment

**Fig. (2):**
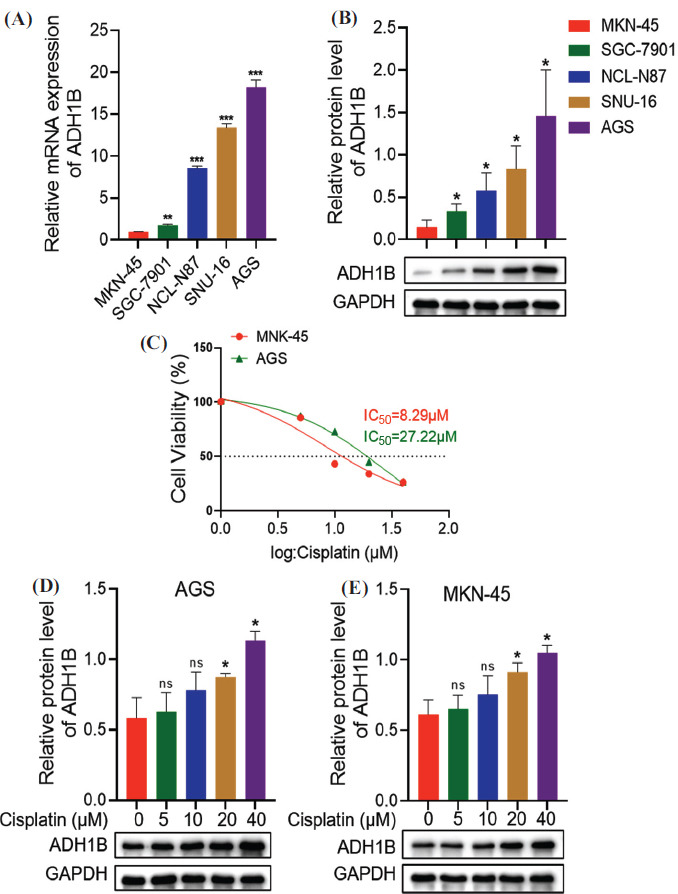
Different expression levels of ADH1B in different gastric cancer cells with different sensitivities to cisplatin. (**A** and **B**) qPCR and WB detection of ADH1B expression in different gastric cancer cells. C: inhibitory effect of cisplatin on different gastric cancer cells. (**D** and **E**) the relationship between the concentration of cisplatin and the expression level of ADH1B in AGS cell and MKN-45 cell. ns: the difference was not significant, *: *p* < 0.05, the difference was statistically significant.

The expression of ADH1B in MKN-45, SGC-7901, NCL-N87, SNU-16, and AGS cells was assessed using qPCR (Fig. **2A**). The results indicated that different gastric cancer cells exhibited varying levels of ADH1B expression, a finding that was corroborated by WB, with results aligning consistently (Fig. **2B**). Consequently, the AGS cell, which displayed the highest ADH1B expression, and the MKN-45 cell, which showed the lowest, were selected for further analysis. The IC50 values for various concentrations of cisplatin on AGS and MKN-45 cells were determined using the CCK8 assay (Fig. **2C**). At all concentrations, MKN-45 cells were lower than AGS cells. Higher IC50 values indicate reduced sensitivity to the drug, indicating that MKN-45 cells are more responsive to cisplatin than AGS cells. In addition, WB was used to evaluate the effects of different cisplatin concentrations on ADH1B expression in MKN-45 cells and AGS cells (Figs. **2D**-**E**). Combining the IC50 of AGS cells and MKN-45 cells, we applied 0 to 40 μM cisplatin to gradient treatment of the cells, and the results showed that the expression of ADH1B in both cells gradually increased with the increase in cisplatin concentration. Based on this, we chose an intermediate concentration of 10μM as the experimental concentration for subsequent cisplatin treatment.

### 3.3 AGS is more Sensitive to Cisplatin after Low Expression of ADH1B

**Fig. (3):**
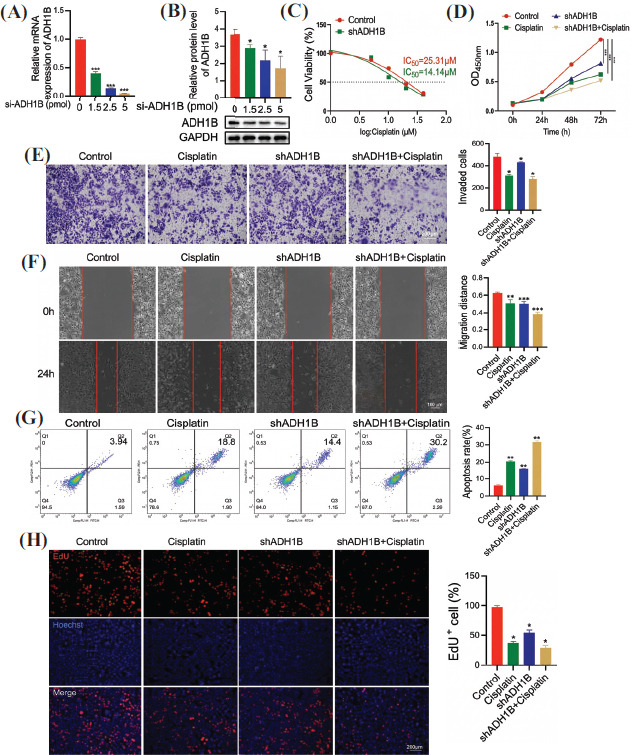
The proliferation, migration, invasion, and apoptosis levels of AGS cells were changed after low expression of ADH1B. (**A-B**) qPCR, WB to verify the silencing efficiency of si-ADH1B. (**C**) Comparison of the inhibitory effects of different concentrations of cisplatin on AGS and AGS cells with low expression of ADH1B (shADH1B). (**D**) CCK8 experiments at different times for the detection of cell viability comparing AGS and shADH1B and the effect of cisplatin on the viability of the two cells. (**E-G**) Comparison of AGS and shADH1B cell function before and after cisplatin treatment. € Assessment of invasion ability; (**F**) Assessment of cell migration ability; (**G**) Level of apoptosis; (**H**) EdU proliferation assay. *: *p* < 0.05, **: *p* < 0.01, ***: *p* < 0.001.

According to Figs. (**3A**-**B**), we found that when the addition level of si-ADH1B reached 5 pmol, the expression level of ADH1B was the lowest, and the silencing efficiency was the best. On this basis, we constructed a shADH1B AGS stable cell line. At different time points following the addition of the same concentration of cisplatin, the activities of the two cell types exhibited more pronounced differences at 48 and 72 hours (Figs. **3C**-**D**). Cisplatin was more effective in inhibiting AGS cells with low ADH1B expression. In the cell invasion assay, the number of cells invading the bottom of the chamber in the AGS group before and after cisplatin treatment was higher than that in the shADH1B group. This indicated that the invasion ability of AGS cells was reduced due to the decreased expression of ADH1B (Fig. **3E**). In the scratch assay, the migratory capacity of AGS cells was reduced after down-regulation of ADH1B expression compared to controls (Fig. **3F**). The apoptosis results showed that the apoptosis rate was decreased in the shADH1B group compared with the control group before the addition of cisplatin. Additionally, following treatment with cisplatin, the rate of apoptosis observed in the shADH1B group was also greater than that seen in the AGS group (Fig. **3G**). EdU using untreated AGS as a control indicated that the proliferation rate of the shADH1B group was diminished compared to that of the AGS group, with cisplatin exerting a stronger inhibitory effect on the shADH1B group (Fig. **3H**). In conclusion, the reduced expression of ADH1B in AGS cells leads to diminished cell viability, along with decreased cell proliferation, migration, and invasion capabilities, and increased sensitivity to cisplatin.

### 3.4 Cisplatin Becomes Less Toxic to MKN-45 Cells After ADH1B Overexpression

**Fig. (4):**
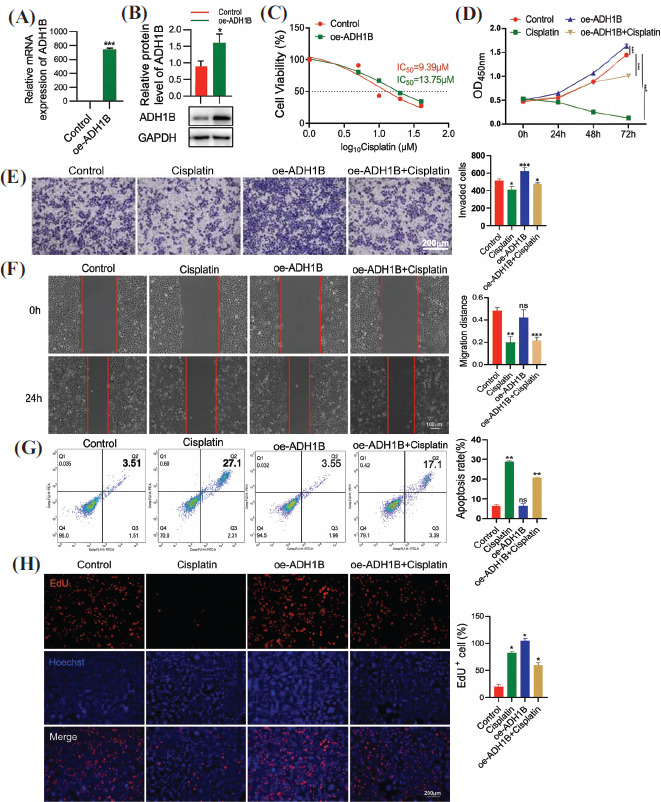
The proliferation, migration, invasion, and apoptosis levels of MKN-45 cells were changed after elevated ADH1B expression. (**A-B**) qPCR and WB assays to compare ADH1B expression in MKN-45 and MKN45 cell overexpressing ADH1B (oe-ADH1B). (**C**) Comparison of the inhibitory effects of different concentrations of cisplatin on the cell viability of MKN-45 and oe- ADH1B cell. (**D**) Cell viability of MKN-45 and oe-ADH1B compared by CCK8 assay at different times and the effect of cisplatin on the cell viability of the two cells. (**E-G**) Comparison of the cell function of MKN-45 and oe-ADH1B cell before and after the treatment of Cisplatin. (**E**) Evaluation of invasive ability; (**D**) Cell migration ability assessment; (**G**) apoptosis level; (**H**) EdU proliferation assay. ns: differences were not significant, *: *P* < 0.05, **: *P* < 0.01, ***: *P* < 0.001.

In contrast, we constructed an oe-ADH1B overexpressing ADH1B using lentiviral transfection to assess changes in the cellular function of MKN-45 following increased ADH1B expression. The findings from qPCR and WB demonstrated that the construction of ADH1B overexpressing MKN-45 cell (oe-ADH1B) was successful (Figs. **4A**-**B**). The CCK-8 assay demonstrated a decreased sensitivity of oe-ADH1B to cisplatin compared to controls (Figs. **4C**-**D**). Compared with the controls, the number of invaded cells in the invasion experiment was significantly higher in the oe-ADH1B group before and after cisplatin treatment than in the control group (Fig. **4E**). In the scratch experiment, the two groups did not show significant differences before cisplatin treatment, but the migration distance of oe-ADH1B after cisplatin treatment elevation was not obvious (Fig. **4F**). Before the treatment with cisplatin, there were no notable differences in apoptosis between controls and oe-ADH1B. However, after cisplatin treatment, the apoptosis rate of MKN-45 cells was higher than that of the oe-ADH1B group (Fig. **4G**). The results of EdU experiments showed that the number of EdU-positive cells was higher in the oe-ADH1B group than in the control group both before and after cisplatin treatment (Fig. **4H**). Collectively, these experiments illustrated that increased ADH1B expression enhances cellular proliferation, invasion, and migration while concurrently reducing sensitivity to cisplatin.

### 3.5 Signal Transduction Pathway Involved in ADH1B Protein

**Fig. (5):**
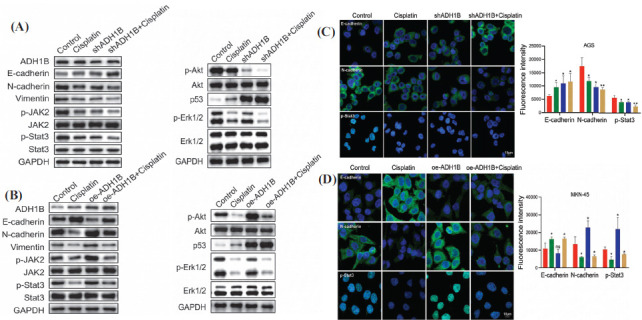
Effect of ADH1B expression on signature proteins and resistance pathway proteins in AGS cell and MKN-45 cell. (**A**) WB detection of signal transduction pathway protein in AGS cells with ADH1B silencing. (**B**) WB detection of signal transduction pathway protein in MKN-45 cells with ADH1B overexpression. (**C**) Immunofluorescence detection of E-cadherin, N-cadherin, and p-Stat3 expressions in AGS cell. (**D**) Immunofluorescence detection of E-cadherin, N-cadherin, and p-Stat3 expressions in MKN-45 cell. Cadherin, N-cadherin, and p-Stat3 expression in AGS cell. (**C**) Immunofluorescence detection of E-cadherin, N-cadherin, and p-Stat3 expression in MKN-45 cell. ns: the difference was not significant, *: *p* < 0.05, **: *p* < 0.01.

We further analyzed the signal transduction pathways involved in ADH1B protein expression. As shown in Fig. (**5**), we detected four iconic proteins, EMT, P53, MAPK, and PI3K/Akt, which are significantly related to the proliferation, growth, migration, invasion, and drug resistance of cancer cells. In the AGS silencing group, compared with the control group, the expression of ADH1B in the shADH1B group was decreased. The expression of E-cadherin increased, the expression of N-cadherin, Vimentin, p-JAK2, and p-Stat3 decreased, the expression of p-Akt, pErk1, and p-Erk2 decreased, and the expression of p53 increased. The comparison of cells in the cisplatin group and shADH1B+Cisplatin group also showed the same trend (Fig. **5A**). In contrast to silencing in the MKN-45 overexpression group, ADH1B expression was increased, E-cadherin expression was decreased, and N-cadherin, Vimentin, p-JAK2, and p-Stat3 expression were increased in oe-ADH1B group compared to the control group. The expressions of p-Akt, pErk1, and p-Erk2 were increased, while the expression of p53 was decreased. After treatment with cisplatin, the trend of protein expression of cells in the oe-ADH1B+Cisplatin group was consistent with that in the oe-ADH1B group (Fig. **5B**). Additionally, the expressions of E-cadherin, N-cadherin, and p-Stat3 in the cell were confirmed through immunofluorescence analysis (Figs. **5C**-**D**), with results consistent with those obtained from WB. The results indicate that ADH1B could lower the sensitivity of gastric cancer cells to cisplatin through the modulation of the EMT pathway and other drug resistance-associated pathways.

### 3.6 Effect of ADH1B on the Tumorigenic Ability of Gastric Cancer Cells and Cisplatin Therapy

**Fig. (6):**
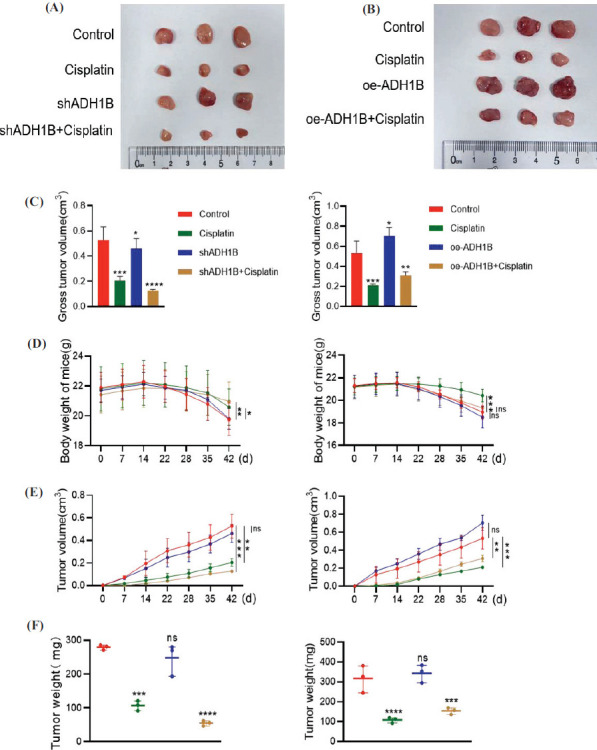
Effects of ADH1B on tumor growth in mice and tumor efficacy of cisplatin. (**A**) Comparison of tumor sizes in AGS cell-loaded mice. (**B**) Comparison of tumor sizes in MKN-45 cell-loaded mice. (**C-F**) AGS cell-loaded mice on the left and MKN-45 cell-loaded mice on the right. (**C**) Total tumor volume. (**D**) Mice body weight. (**E**) Tumor volume of the change. (**F**) tumor weight. ns: difference is not significant, *: *p* < 0.05, **: *p* < 0.01, ***: *p* < 0.001, ****: *p* < 0.0001.

In the tumorigenesis experiment (Fig. **6**), shADH1B tumors without cisplatin treatment were slightly smaller than those in the control group. After cisplatin treatment, the tumors in the shADH1B group significantly decreased further (Fig. **6A**), and the weight, tumor volume, and total weight of the mice were reduced, while the results of overexpression were the opposite (Figs. **6B**-**6F**).

**Fig. (7):**
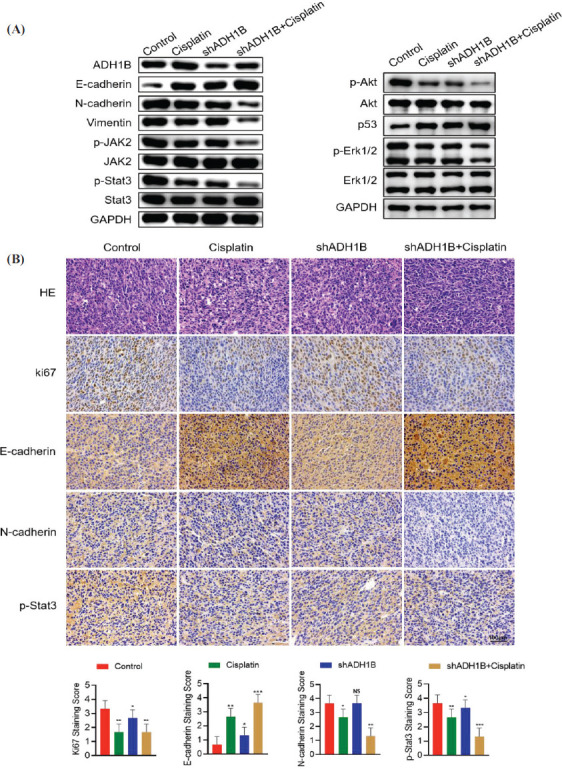
ADH1B silencing changes tumor tissue structure and signal transduction pathway protein expression. (**A**) WB detection of signal transduction pathway protein in tumor tissue with ADH1B silencing. (**B**) Results of HE staining and immunohistochemical detection of Ki67, E-cadherin, N-cadherin, and p-Stat3 expression in tumor tissues of AGS and shADH1B cell mouse models. *: *p* < 0.05, **: *p* < 0.01, ***: *p* < 0.001.

**Fig. (8):**
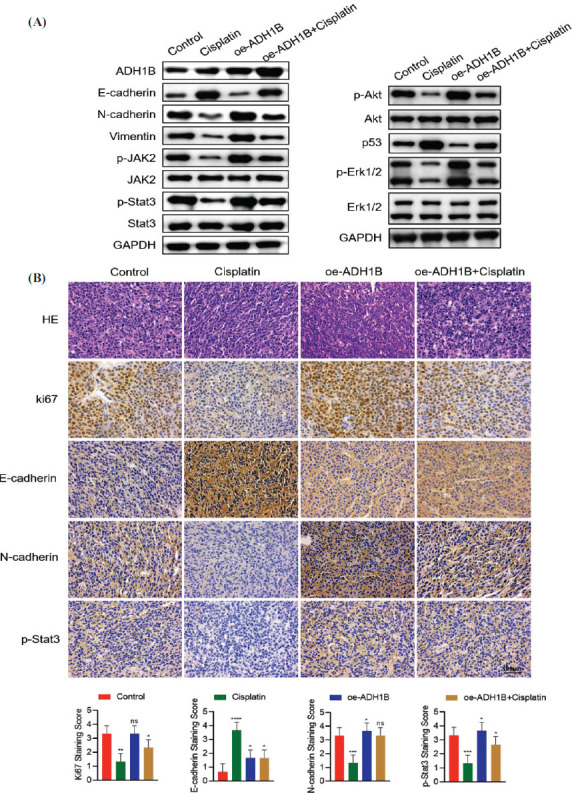
ADH1B overexpression changes tumor tissue structure and signal transduction pathway protein expression. (**A**) WB detection of signal transduction pathway protein in tumor tissue with ADH1B overexpression. (**B**) Results of HE staining and immunohistochemical detection of Ki67, E-cadherin, N-cadherin, p-Stat3 expression in tumor tissues of MKN-45 and oe-ADH1B cell mouse models. *: *p* < 0.05, **: *p* < 0.01, ***: *p* < 0.001.

The results of the signal transduction pathway detected by WB in the tissue were consistent with those of cells (Fig. **7A**). The results of HE staining showed that the tumor cell density in the shADH1B group was reduced, and the tissue was relatively loose (Fig. **7B**). Immunohistochemical results showed that E-cadherin expression increased in shADH1B tumor tissues, while Ki 67, N-cadherin, and p-Stat3 expression decreased (Fig. **7B**). On the contrary, the signal transduction pathway results detected by WB in the overexpressed tumor tissues showed that EMT, P53, MAPK, and PI3K/Akt were also affected by the expression level of ADH1B (Fig. **8A**). Compared with the control group, after overexpression of ADH1B, HE staining showed that the tumor tissue was more dense and the cells were more chaotic (Fig. **8B**). Immunohistochemical results showed that the expression of E-cadherin was decreased in oe-ADH1B tumor tissue, while the expression of Ki 67, N-cadherin, and p-Stat3 was increased (Fig. **8B**). These results indicated that ADH1B overexpression in tumor tissues promoted tumor formation of gastric cancer cells by affecting EMT, P53, MAPK, and PI3K/Akt pathways and decreased the therapeutic effect of cisplatin.

## CONCLUSION

We demonstrated that ADH1B promotes gastric carcinogenesis and confers resistance to cisplatin through the EMT pathway. Specifically, ADH1B expression influences the levels of EMT pathway signature proteins as well as several proteins associated with resistance pathways. This discovery improves our comprehension of the role of ADH1B in the development of gastric cancer and provides novel perspectives on the mechanisms that contribute to cisplatin resistance in gastric cancer cells. Consequently, ADH1B might serve as a new target for overcoming resistance to cisplatin and curtailing the advancement of gastric cancer.

## 4 DISCUSSION AND STUDY LIMITATIONS

Cisplatin is the primary chemotherapeutic agent for patients with gastric cancer, particularly in advanced stages. D. Huang and colleagues [21] discovered that the increased expression of HER2 in gastric cancer cells resistant to cisplatin resulted in morphological alterations similar to EMT, indicating a connection between cisplatin resistance and EMT in these cancer cells. Furthermore, ADH1B has been associated with multiple cancer types, such as head and neck cancer [22] and cancers of the upper gastrointestinal tract [23]. ADH1B can also be used as a prognostic marker and target marker for chemotherapy in GC [24].

E-cadherin, N-cadherin, and vimentin are key genes linked to the EMT pathway [25]. Tumor cells that undergo EMT display changes in morphology and functionality, marked by a decrease in epithelial markers, including E-cadherin, alongside a rise in mesenchymal markers, such as N-cadherin and vimentin [26-27]. There are fewer studies on the relationship between the ADH1B gene and EMT, and the link between the two is not yet known. However, it is clear that ADH1B is associated with the development of GC, while EMT promotes GC progression. We hypothesize that there is an association between ADH1B and EMT.

Our analysis of the correlation between ADH1B and E-Cadherin/VIM indicates that ADH1B is significantly associated with the genes that define EMT. Furthermore, our research revealed that the expression levels of ADH1B vary among different gastric cancer cells, and sensitivity to cisplatin may also vary accordingly. We selected MKN-45, which exhibited the lowest ADH1B expression among the tested cells, and AGS, which displayed the highest expression, to compare their sensitivity to different concentrations of cisplatin. The IC50 values for both cells decreased gradually with increasing cisplatin concentration. However, the IC50 value for AGS was consistently higher than that of MKN-45 at each concentration tested. This suggests an inverse relationship between ADH1B expression in tumor cells and sensitivity to cisplatin. Furthermore, as cisplatin concentration increased, ADH1B expression also increased. Therefore, we determine that the sensitivity of gastric cancer cells to cisplatin is linked to their inherent expression levels of ADH1B and that the expression of ADH1B increases with higher concentrations of cisplatin, indicating a reciprocal interaction between the two.

To eliminate potential differences between various cells, we constructed oe-ADH1B and shADH1B. The AGS cell, characterized by low ADH1B expression, exhibited increased sensitivity to cisplatin alongside diminished proliferation, migration, and invasion capabilities. Conversely, MKN-45 cells exhibiting higher levels of ADH1B expression showed decreased sensitivity to cisplatin, along with improved capabilities in proliferation, migration, and invasion. The expression of ADH1B significantly influenced the functional alterations of tumor cells. Notably, shADH1B cells displayed some degree of apoptosis under normal culture conditions. The growth of oe-ADH1B cell was significantly higher compared to that of the MKN-45 cell. This suggests that reduced expression of ADH1B may lead to increased apoptosis in tumor cells, whereas higher expression promotes their proliferation.

Cisplatin is initially effective in killing tumor cells. However, resistance to the drug gradually develops in later stages. Additionally, ADH1B promotes tumorigenesis and metastasis, yet no study has clarified whether there is an association between ADH1B and tumor resistance to cisplatin. In light of literature indicating that gastric cancer cell resistance to cisplatin is associated with EMT, the expression levels of EMT signature proteins, including E-cadherin, N-cadherin, and Vimentin, were examined alongside the resistance pathways involving p-JAK2, JAK2, p-Stat3, and Stat3. It was discovered that the pathways related to p-JAK2, JAK2, and p-Stat3 were linked to cisplatin resistance, EMT, and the advancement of gastric cancer development [28-33]. The expression of ADH1B varied, and the epitopes of all proteins, except Stat3, differed. Previous research indicated that increased levels of E-cadherin are related to a reduced metastatic capability in tumor cells, whereas decreased expression heightens their metastatic potential [34]. Conversely, higher expression levels of N-cadherin, vimentin, p-JAK2, and p-Stat3 indicate greater migratory and invasive capabilities of tumors, as well as increased resistance to chemotherapeutic agents [35-37]. Our results demonstrated that lower expression levels of ADH1B correlate with higher expression of E-cadherin and increased sensitivity to cisplatin. Conversely, higher expression of ADH1B is associated with elevated levels of N-cadherin, Vimentin, p-JAK2, and p-Stat3, leading to more pronounced resistance to cisplatin. ADH1B influences the functionality of gastric cancer cell and their resistance to cisplatin through the EMT pathway. *In vivo* studies indicated that tumors with high ADH1B expression exhibited more significant growth, and the therapeutic efficacy of cisplatin treatment was inferior compared to tumors with lower ADH1B expression. Furthermore, results from HE staining and immunohistochemical analysis of Ki67 corroborated that tumor tissues with lower ADH1B expression exhibited reduced cisplatin-related pathology. The levels of expression for E-cadherin, N-cadherin, and p-Stat3 were consistent with findings from previous investigations into related pathways [39-41].

## Data Availability

The datasets used and/or analyzed during the current study are available from the corresponding author upon reasonable request.

## LIST OF ABBREVIATIONS

ADH1B: Alcohol Dehydrogenase 1B
CCK-8: Cell Counting Kit-8
EDU: 5-ethynyl-2’-deoxyuridine
EMT: Epithelial-mesenchymal Transition
GC: Gastric Carcinoma
HE: Hematoxylin-eosin Staining
IC50: The Half Maximal Inhibitory Concentration
MAPK: Mitogen-activated Protein Kinase
PI3K: Phosphatidylinositol 3-kinase
WB: Western Blot
